# Enabling artificial photosynthesis systems with molecular recycling: A review of photo- and electrochemical methods for regenerating organic sacrificial electron donors

**DOI:** 10.3762/bjoc.19.88

**Published:** 2023-08-08

**Authors:** Grace A Lowe

**Affiliations:** 1 van ’t Hoff Institute for Molecular Sciences (HIMS), Universiteit van Amsterdam (UvA), Science Park 904, Amsterdam, 1098 XH, The Netherlands

**Keywords:** artificial photosynthesis, photocatalysis, redox couple, sacrificial electron donor, solar fuels

## Abstract

This review surveys advances in the literature that impact organic sacrificial electron donor recycling in artificial photosynthesis. Systems for photocatalytic carbon dioxide reduction are optimized using sacrificial electron donors. One strategy for coupling carbon dioxide reduction and water oxidation to achieve artificial photosynthesis is to use a redox mediator, or recyclable electron donor. This review highlights photo- and electrochemical methods for recycling amines and NADH analogues that can be used as electron donors in artificial photosynthesis. Important properties of sacrificial donors and recycling strategies are also discussed. Compounds from other fields, such as redox flow batteries and decoupled water splitting research, are introduced as alternative recyclable sacrificial electron donors and their oxidation potentials are compared to the redox potentials of some model photosensitizers. The aim of this review is to act as a reference for researchers developing photocatalytic systems with sacrificial electron donors, and for researchers interested in designing new redox mediator and recyclable electron donor species.

## Introduction

Artificial photosynthesis research has resulted in the discovery and creation of incredible chemical systems and materials. The ultimate goal is to harness energy from the sun and use it to transfer electrons and protons from water onto carbon dioxide and create molecules to replace fossil fuels [1–2]. However, when developing the components of artificial photosynthesis systems species other than water are consumed to provide these electrons and protons [3–4]. Ideally these sacrificial donors would be replaced with redox mediators, regenerated using water, or form stable, commercially valuable oxidation products. However, common sacrificial electron donors, such as triethylamine, breakdown after oxidation which prevents regeneration [3,5]. Furthermore, there have also been studies where systems have been developed with sacrificial donors that interact and actually change the reactivity of the system [5–6]. Replacing these sacrificial donors becomes more challenging in these scenarios. There is currently a steadily growing body of research investigating recycling of sacrificial electron donors. Meanwhile, advances in other fields have resulted in a vast array of alternative redox mediators and recyclable electron donors to explore.

Sacrificial donors have been used in artificial photosynthesis research to model different key photosynthesis processes. In plants, photosynthesis is a complex process where light-harvesting reactions are carried out in two photosystems to split water and recycle NADH and ADP. NADPH and ATP are then consumed in the Calvin cycle to reduce and fixate carbon dioxide [7]. Quite sensibly, many research groups investigating artificial photosynthesis develop components and systems for water splitting and carbon dioxide reduction separately before they or others seek to combine them. This modular approach is hugely beneficial because it can allow coupling of different reaction systems [2,8]. This strategy also works well for developing photoelectrochemical systems where the oxidation and reduction can be confined at separate electrodes.

When developing reactions for carbon dioxide reduction in a modular fashion isolated from water splitting, sacrificial electron donors are used as an electron source to act as a placeholder for NADPH and the water-splitting reaction. To recouple water splitting and carbon dioxide reduction the sacrificial donors need to be replaced by redox mediators. A redox mediator is a compound or material that shuttles electrons from one species to another through a series of chemically reversible reduction and oxidation reactions. In contrast, a sacrificial electron donor is a species that is oxidized to reduce another species and is consumed rather than regenerated. If a redox mediator is not re-reduced, then it is functioning as a sacrificial electron donor. However, not all sacrificial donors used to develop carbon dioxide reduction systems can be used as redox mediators because during oxidation they form products that cannot be regenerated. As a result, a different redox mediator compound or material is needed to couple the carbon dioxide reduction system to water splitting. That said, to successfully reduce a molecule there must always be a sacrificial electron donor or stoichiometric reductant. In photosynthesis the sacrificial donor is water, and the byproduct is oxygen.

Sacrificial electron donors are usually small organic molecules which are used in large quantities and need to be cheap. This often means they are less optimized than expensive catalysts and dyes. However, systems that employ redox mediators can use lower concentrations of more expensive species. For example, inorganic Z-schemes have used cobalt complexes and polyoxometalates to shuttle electrons between water oxidation and carbon dioxide reduction photocatalysts [2,4]. However, the photocatalysts of these systems are usually first developed separately with sacrificial electron donors. Other methods for bringing together the 2 halves of artificial photosynthesis include photoelectrochemical cells and artificial leaves, single molecule/particle photocatalysts, photovoltaic-powered electrochemical cells, and biophotoelectrochemical cells [8–9]. Some of these systems, like natural photosynthesis, are decoupled; the water oxidation and carbon dioxide reduction occur at different catalytic centers (locations) and in some cases at different times [8]. Decoupling is generally facilitated by the accumulation of charge or reacted species that can be stored. In artificial photosynthesis decoupling is also possible by storing reduced redox mediators, or regenerated electron donor species if they cannot simply be re-reduced. Not all approaches to carbon dioxide reduction in artificial photosynthesis require small organic sacrificial donors or mediators. This review focuses on regeneration and recycling of small organic molecules that can be used as sacrificial donors in photochemical carbon dioxide reduction.

A large scale decoupled, or macro, model of photosynthesis is electrochemical carbon dioxide reduction powered by renewable energy sources. Electrochemical carbon dioxide reduction has been commercialized. Specifically, the company Twelve are making large advances in the electrolysis of carbon dioxide to carbon monoxide. Their contracts started with materials and have now expanded to fuels [10]. However, industrial electrochemistry either requires a dedicated power source, or plugging into a national electricity grid. In countries like the UK, the competition to install new batteries and renewable energy sources appears to have created an increase in demand and waiting times for installing grid connections [11–12]. This is potentially a large barrier to fast adoption and scaling of purely electrochemical methods. However, in the short term this means that there is an opportunity, or unmet need, for simple photochemical systems that generate storable fuel/feedstocks without a grid connection or similar infrastructure. Consequently, this potential gap in the solar fuel/feedstock market makes it more important to replace unrecyclable sacrificial donors in molecular systems. Recently researchers have been looking at replacing traditional sacrificial donors with food and plastic waste [13]. This exciting new field of photoreforming does not involve regeneration of donors or the use of redox mediators, so it will not be covered in this review.

Most molecular components for photoreduction catalysis are not being developed or optimized with sacrificial electron donors that can be recycled. This means that the conditions must be reoptimized if redox mediators or other recyclable donors need to be used to couple the reduction to water oxidation or other reactions. Furthermore, the performance of the molecular photosensitizer and catalyst combinations developed are often very dependent on the properties of the sacrificial donors. This review has two aims: 1. Highlight work being done to recycle sacrificial donors used in photoreduction catalysis for artificial photosynthesis. Specifically, organic electron or hydride donors usually applied in molecular photocatalysis. 2. Survey the literature from different fields and present a sample of potentially recyclable electron donors for artificial photosynthesis, alongside the properties important for comparing the suitability of different donors. Such a resource should hopefully help to increase adoption of recyclable donors when developing photoreduction systems for artificial photosynthesis, as well as highlight gaps where new donor compounds are required.

## Review

### What makes a good sacrificial electron donor?

**Figure 1:**
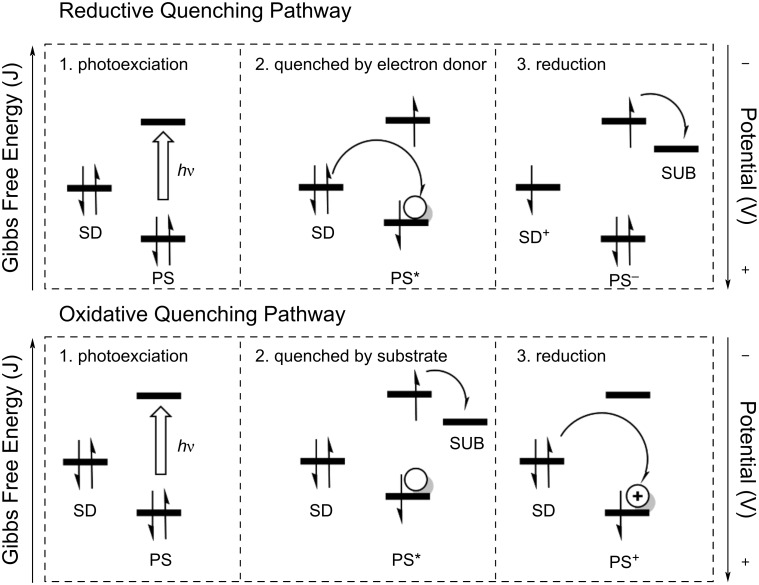
Diagram comparing the two reaction pathways for sacrificial electron donors (SD) in photocatalyzed reductions: reductive quenching and oxidative quenching. Energy levels of the photosensitizer (PS), sacrificial electron donor (SD), and substrate/catalyst (SUB) have been sketched relative to each other on an energy axis. Electron transfer between energy levels is indicated by half-arrows, and photoexcitation is indicated by arrows labelled *h*ν. The numbers indicate the order of electron transfer and orbital population is indicated by short arrows and circles. This figure was created to illustrate ideas communicated in references [3] and [18].

Before exploring sacrificial electron donor recycling, it is important to understand what chemical properties and behavior make efficient sacrificial electron donors. Sacrificial electron donors reduce either photoexcited or photooxidized photosensitizers in systems for carbon dioxide reduction (see Figure 1). Reductive quenching of the photosensitizer occurs when the sacrificial donor reduces the photoexcited photosensitizer (reductive quenching pathway). Regeneration of photooxidized photosensitizers occurs when the excited dye is first oxidatively quenched by a substrate or catalyst and then reduced by the sacrificial donor (oxidative quenching pathway). In the presence of protons, proton donors, or oxidized donor species with a low p*K*_a_, a proton-coupled electron transfer (PCET) can take place [14–15]. PCET reactions are important in artificial photosynthesis research not only because they occur in biological photosynthesis but also because the PCET can circumvent unstable one electron-reduced intermediates. This makes PCET mechanisms well-suited for complex multielectron reactions required to transfer electrons and protons from water onto carbon dioxide [16]. Excited-state PCET, which is of particular interest for interactions between hydrogen atom or hydride-donating sacrificial donors, has recently been reviewed in detail by Dempsey and co-workers [17].

To select an effective sacrificial electron donor, at least four properties need to be considered: solubility in the chosen solvent, absorption spectrum, oxidation potential, and the reversibility of the sacrificial donor oxidation. As the sacrificial donor is a reactant in the photoreduction reaction of carbon dioxide, it needs to be highly soluble in the solvent used. It also needs to have a low absorption in the visible region to prevent side reactions and allow the photosensitizer to absorb as much light as possible. The oxidation potential of the sacrificial electron donor must be less positive than the reduction potential of the excited or oxidized photosensitizer for quenching or regeneration to occur. As shown in Figure 1, the scale of electrochemical potential measured in volts is an inverted scale of free energy. Therefore, it is thermodynamically favorable for electrons to be transferred from higher energy sacrificial donor orbitals with less positive oxidation potentials to lower energy orbitals on photooxidized or photoexcited photosensitizers with more positive potentials.

Cyclic voltammetry can be used to measure ground-state redox potentials by varying the potential at a working electrode with respect to a reference electrode and measuring the current response [19]. In photochemical carbon dioxide reduction research, this technique is used to measure the reduction potential of the oxidized photosensitizer and the oxidation potential of the electron donor. However, the reduction potential of the photoexcited photosensitizer is usually estimated using the Rehm–Weller equation and the ground-state redox potentials of the photosensitizer [18,20], or simulated [21–22]. There are actually methods for directly measuring the excited-state redox potentials of a photosensitizer, such as photomodulated voltammetry but they often require a more elaborate experimental setup [23]. It is important to note that unless special electrodes (ultra microelectrodes) or cells are used, voltammetric measurements of redox potentials require an ionically conductive salt to be added to the solution [19].

Oxidation and reduction potentials can vary with factors such as pH [14–15] and solvent polarity [24–25]. Hence, it is important when considering new reagents and catalysts to only compare potentials measured in conditions as close to the photocatalytic conditions as possible. For instance, quinones have 2 one-electron reductions in aprotic media and one two-electron reduction at a shifted potential in aqueous media [26]. As a demonstrative example concerning quenching, Schulz and co-workers only detected the 2-electron-reduced product of the Cu(I*)* 4*H*-imidazolate complex when it was irradiated in aprotic media in the presence of the sacrificial donor *p*-(dimethylamino)toluene (DMT) [27]. However, voltammetry in aprotic media indicated that a stable one-electron-reduced product should have been formed. Adding an inert ammonium salt to the voltammetry experiment mimicked the associative behavior of oxidized DMT and recreated the 2-electron reduction that occurred during photocatalysis. Cyclic voltammetry carried out in standard conditions for mimicking an acetonitrile system had not been close enough to the catalytic conditions to get the required redox potentials.

The difference between the oxidation potential of a sacrificial donor and the reduction potential of the excited or oxidized photosensitizer is the driving force for the electron transfer and photosensitizer regeneration. This driving force determines the rate of electron transfer from the electron donor to the photosensitizer which regulates the amount of photosensitizer available to harvest light energy and controls the turnover rate (often measured as turnover number), and productivity of the entire photocatalytic system. In order for the photoreduction system to work, either the reductive quenching or the oxidative quenching of the photosensitizer must be faster than the decay of the photosensitizer’s excited state. Hence, Stern–Volmer plots derived from quenching measurements and photosensitizer excited-state lifetimes measured by transient spectroscopy are crucial to understanding and optimizing the system as a whole [18].

Recently, Kientz et al. demonstrated that the dark regeneration of oxidatively quenched photosensitizers can also limit the whole system in photochemical carbon dioxide reduction [28]. In the study, the driving force for reduction of an oxidized sensitizer was varied by using a series of photosensitizers with tunable oxidation potentials. They found that the turnover of the carbon dioxide reduction system was limited by the rate of the photooxidized sensitizer re-reduction.

In their review of sacrificial electron donors for solar fuels, Pellegrin and Odobel noted that an effective sacrificial donor must be irreversibly oxidized into inert molecules [3]. This prevents side reactions and allows the accumulation of the oxidized species and almost complete consumption of the sacrificial donor. However, thermodynamically irreversible does not mean that the sacrificial donor must break down into unrecyclable fragments like triethylamine (TEA). An irreversible electron transfer is slow and mitigates geminate recombination and, in principle, if there is no proceeding chemical step, the product can be re-reduced if an appropriate reducing agent or potential is applied. A reversible electron transfer followed by a fast chemical step, such as dimerization of oxidized dithiolates to sulfides, or a separate deprotonation event can also result in similar behavior. Gimeno et al. highlighted this when they designed novel benzimidazole (BIH) donors for the photoreduction with [Cu(dipp)_2_]^2+^ photocatalysts [29–30]. They contrasted their work to a previous study by Cunningham and McMillin who used a similar photocatalyst to systematically study ferrocene derivatives as sacrificial donors [31]. The ferrocene derivatives reductively quenched the photosensitizer but could not accumulate as effectively as the BIH derivatives used by Gimeno et al. [29,31]. In contrast, Z-schemes that employ redox mediators can use compounds such as cobalt bipyridine complexes which undergo fast reversible electron transfer reactions [2,4,8]. Z-schemes require a steady state concentration of both oxidized and reduced redox mediator species to allow an efficient shuttling of electrons between photocatalysts. Unlike photocatalysis with unrecycled sacrificial donors, the reaction does not depend on the total consumption of the donor. Z-schemes are generally achieved by transfer of electrons to semiconductor particles with photocharged interfaces that can accumulate charge and provide a potential gradient to prevent significant recombination. These features make reversible redox couples suitable donors for Z-schemes when they are not necessarily the most efficient sacrificial donors for other photocatalysis schemes.

### Recycling sacrificial reagents literature highlights

In 2011, Carpenter and co-workers published a study in which they used a cyclic tertiary amine sacrificial donor that they had designed to be regenerated [32]. This was significant because instead of replacing amine sacrificial donors with redox mediators, this paper proposed an alternate strategy of an ex-situ regeneration of organic donors. The authors designed and synthesized a tertiary amine that could be oxidized and rehydrogenated. They used their amine to successfully replace triethylamine in a photocatalytic carbon dioxide reduction reaction from the literature and proved that the recyclable byproduct was formed. In another experiment, the team chemically oxidized the sacrificial donor and regenerated it by hydrogenation.

**Figure 2:**
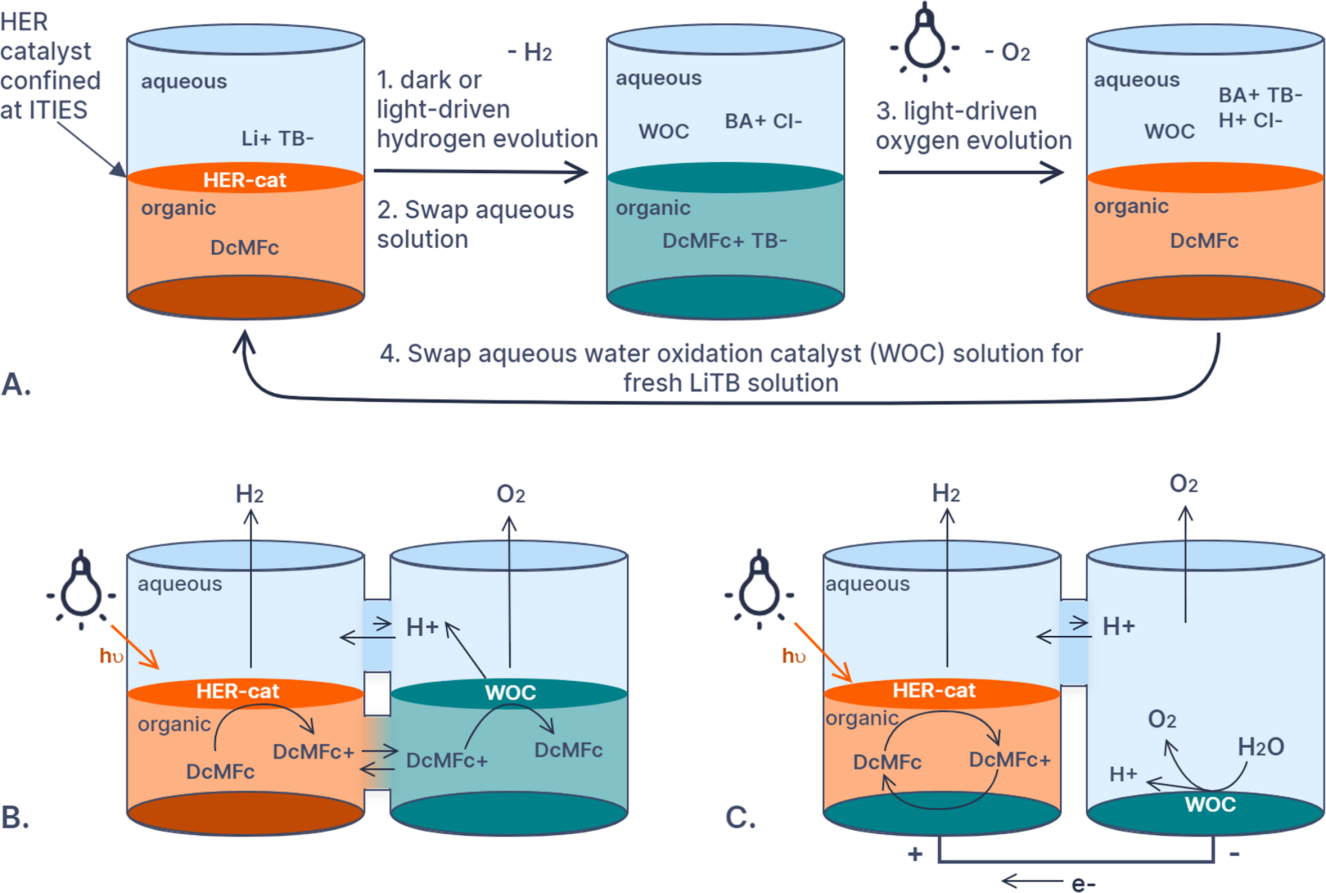
Diagram showing water-splitting systems developed by Girault, Scanlon, and co-workers that employ interfaces between two immiscible electrolyte solutions (ITIES) to enable recycling of the decamethylferrocene (DcMFc) sacrificial donor [35]. A. Photocharging and recycling of DcMFc by swapping aqueous solutions between mixtures containing a water-oxidation catalyst (WOC) and a hydrogen-evolution reaction catalyst (HER-cat). B. Photorecycling of DcMFc using ITIES to separate charge and catalysts for water splitting. This scheme can be described as a decoupled Z-scheme. C. Photogalvanic cell where the potential is established by photocharging of the ITIES. DcMFc is electrochemically recycled in-situ and water oxidation happens at the counter electrode. Note, that water is omitted for clarity in A, B, and C. The conductive salts are omitted for clarity in B and C. The lipophilic tetrakis(pentafluorophenyl)borate (TB^−^) and bis(triphenylphosphoranylidene)ammonium (BA^+^) salts were crucial to allow charge transfer at the ITIES.

Carpenter and co-workers briefly discussed phase separation to enable sacrificial donor recycling by improving the recovery of the oxidized donor [32]. This idea was central to the works published by Girault, Scanlon and co-workers on photocatalytic water splitting [33–35]. They used the redox mediator decamethylferrocene (DcMFc) in biphasic systems and semi-immobilized their photosensitizers and catalysts at interfaces between two immiscible electrolyte solutions (ITIES). This facilitated the in-situ redox mediator recycling and separation. The authors actually employed this strategy using 3 different reactor configurations (Figure 2). The first and simplest ex-situ photorecycling method involved adding and extracting 2 aqueous phases containing the catalysts and reactant to discharge and charge the donor-containing organic phase (Figure 2A) [34]. The first solution contained an organic lithium salt and a hydrogen-evolution catalyst which could generate hydrogen via a light-driven or dark process. The second solution contained an organic chloride salt and a water oxidation photocatalyst which re-reduced DcMFc and evolved oxygen. The same reaction scheme was used but in a modified H-cell (Figure 2B) [35]. Both catalysts were confined at the ITIES in two separate chambers and the redox mediator diffused between the two cells via the organic phase. The protons for hydrogen evolution migrated and diffused via the aqueous layer. The photoelectrochemical recycling was also studied in a system with one ITIES where the photocatalyst was immobilized and water oxidation was carried out at an electrode in the aqueous layer (Figure 2C). This electrode was connected to a counter electrode in the non-aqueous half-cell where DcMFc was re-reduced [35]. The potential difference in this reactor was generated by the photocatalyst excitation at the ITIES and not at the electrode, making it a photogalvanic cell. A subtle but important feature of these systems is that the partition of the electrolyte salt between the two solutions imposed an electrochemical bias that decreased the rate of chemical recombination between the two phases. Rastgar and Wittstock studied this phenomenon and the mechanistic details of photo- and electrocatalysis at ITIES with modified scanning electrochemical microscopy [36–37]. In nature, chemical gradients and phase separation are maintained by compartmentalization in liposomes, micelles, and vesicles rather than at interfaces such as ITIES. Artificial photosynthesis systems are being designed to mimic this behavior and recently the field of artificial photosynthesis using liposomes was thoroughly reviewed with special attention paid to donors and redox mediators [38]. Species such as methyl viologen were employed as redox mediators in some of the systems reviewed, however, the regeneration still required consumption of species other than water, such as EDTA.

Carpenter and co-workers also proposed, but did not test, recycling their amine with electrochemistry and light [32]. They cited a work by Itoh et al. who modified a proton exchange membrane electrolyzer with a Rh–Pt catalyst to generate hydrogen from water to hydrogenate benzene to cyclohexane in one reactor [39]. Itoh and co-workers were studying electrochemical hydrogenation for LOHCs rather than the regeneration of sacrificial donors. A lot of small organic compounds have been considered for electrochemical hydrogenation for LOHCs but many do not have the required oxidation potentials to be sacrificial donors [40]. More recently, other groups have published the electrochemical hydrogenation of carbonyl compounds using more earth-abundant electrocatalysts. For instance, Siewert and co-worker used a manganese complex as an electrocatalyst for the chemoselective carbonyl hydrogenation [41]. Behrouzi et al. reported the electrochemical hydrogenation of carbonyl and amido compounds using nickel electrodes and water as the proton and electron source [42]. Furthermore, the carbonyl and amido compounds used in these electrochemical hydrogenation studies are more structurally similar to organic sacrificial donors than cyclohexane. Although these papers were not demonstrating the recycling of sacrificial donors, they both demonstrate that electrochemical hydrogenation could be an extension of artificial photosynthesis for the production of solar fuels and feedstocks [41–42]. There is also a large body of work using simple alcohols as a proton and electron source in electrochemical hydrogenations [43]. This could also evolve into an extension of artificial photosynthesis if the alcohols used as donors are generated by artificial or natural photosynthesis (i.e., photosynthetic bacteria).

NADH and NADH-analogues are the subject of most studies for recycling sacrificial donors. NADH has been electrochemically recycled but a careful control of pH is required to prevent dimerization reactions [44–45]. For example, Glusac and co-workers recycled BIH and acridine analogues using platinum electrodes in acetonitrile with proton donors [45]. They carefully calculated the p*K*_a_H of their proton donors and NADH analogues to control the PCET and to prevent side reactions. In another interesting example, NADH was recycled at a copper electrode in aqueous buffers and NADH was found to be more stable in a tris buffer rather than phosphate [44]. Instead of using the regenerated NADH in a photocatalytic system, this team actually used an enzyme to consume the regenerated NADH and check its viability.

Robert and co-workers recycled the NADH analogue 1,4-BNAH using different photosensitizers and cobalt catalysts in an acetonitrile/water mixture [46]. They used combinations of deuterated solvents and ^1^H NMR spectroscopy to confirm that water was the main source of the protons for the regeneration. Furthermore, they successfully replaced ruthenium photosensitizers with organic dyes so that the system used predominantly earth-abundant materials. The recycling of NADH analogues has been carried out using precious metal complexes, such as [CpRh(bpy)(H_2_O)]^2+^ [47]. This rhodium complex was adhered to a photoelectrode in a photoelectrochemical cell which also contained a second photoelectrode functionalized with a set of enzymes [47]. The enzymes reduce carbon dioxide to methanol and consumed NADH which was then recycled at the photoelectrode functionalized with the rhodium complex. The overall electron donor in this work was water which makes it an excellent example of in-situ recycling. Kuk et al. also noted that [CpRh(bpy)(H_2_O)]^2+^ could slowly produce formate, a key biocatalytic intermediate, in the absence of the enzymes but they validated their system by proving that overall the formate production and conversion to methanol by the biocatalytic enzyme cascade far outcompeted any side-reactions.

Ishitani and co-workers introduced BIH analogues based on the *N*,*N*’-dimethyl-2-phenylbenzimidazole scaffold as more efficient alternatives to NADH-derived electron donors for carbon dioxide reduction photocatalysis [48]. Consequently, BIH analogues are becoming increasingly popular sacrificial electron donors in artificial photosynthesis [3,29,48]. Glusac and co-workers published studies on the photochemical recycling of BIH analogues [49]. They used an organic photocatalyst to reduce the oxidized benzimidazole 1,3-dimethyl-2-(2,4,6-trimethoxyphenyl)-2*H*-benzimidazole (BIM) twice in the presence of various acids [49]. This system is completely metal-free and uses one photocatalyst rather than separate sensitizer and catalyst species. However, the electron source for the reductions was a thiolate sacrificial donor and not water. Thiolates are used as redox mediators in other systems such as dye-sensitized solar cells [50–51].

**Figure 3:**
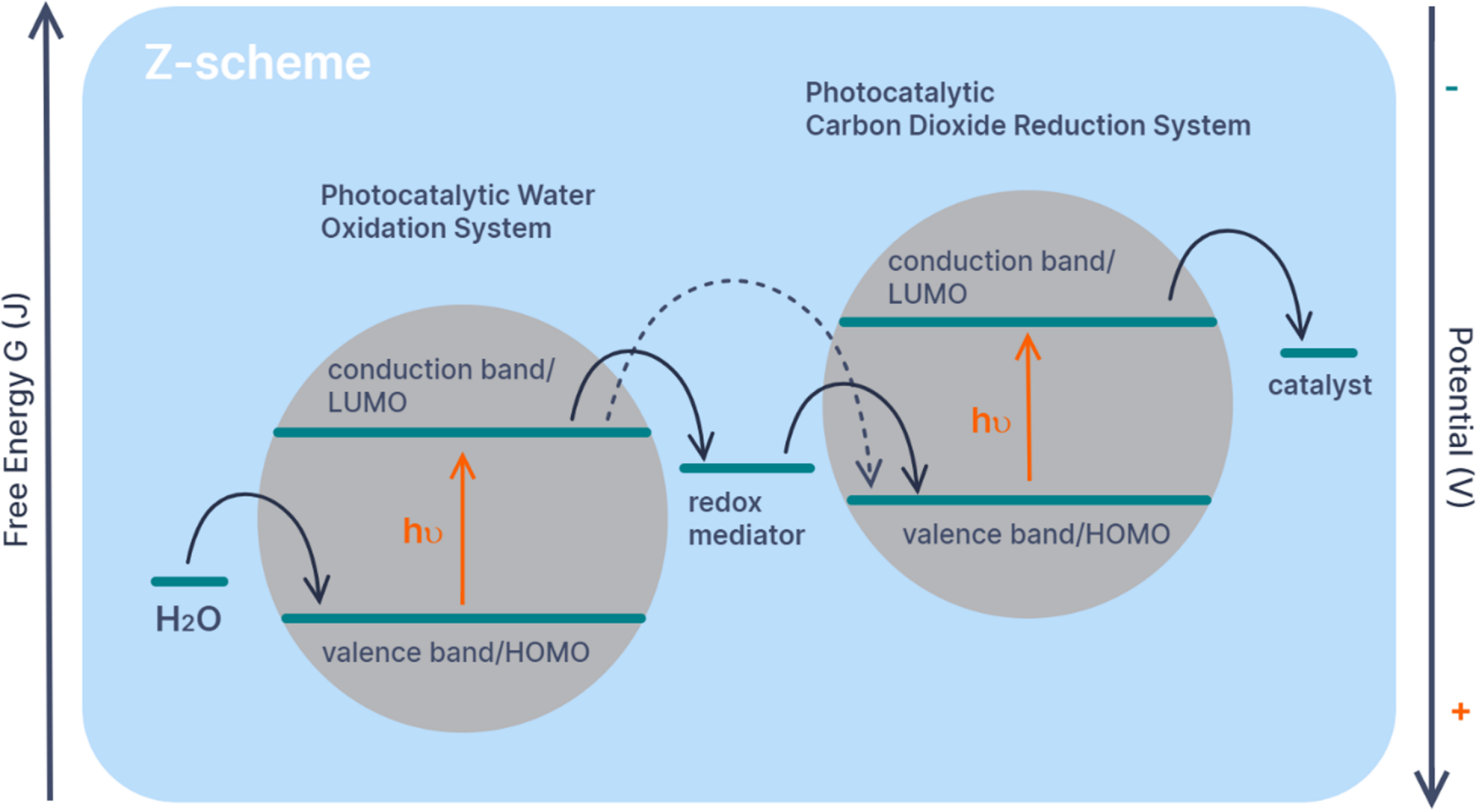
Diagram illustrating the transfer of electrons in a photocatalytic particulate suspensions Z-scheme for carbon dioxide reduction. The dotted arrow indicates the transfer of electrons between the two photocatalytic systems in the absence of a redox mediator, for example, when two systems are anchored together close enough to permit successful electron transfer. The redox mediator can also be replaced by immobilizing the photocatalytic systems on conductive materials or electrodes in an electrochemical cell.

As discussed, if a sacrificial donor is recycled in-situ it becomes a redox mediator. In artificial photosynthesis redox mediators are most commonly employed in Z-schemes. A Z-scheme describes the combination of two photocatalytic systems, one for photooxidation and one for photoreduction, with their energy levels arranged so that they operate together to transfer electrons from one substrate to another (Figure 3). Artificial Z-schemes were used for photochemical water splitting before carbon dioxide reduction and are mainly being investigated using particulate semiconductor photocatalyst composites, such as decorated quantum dots [4]. Most of the particulate Z-schemes use inorganic rather than organic redox mediators [2]. Interestingly, soluble redox mediators can be replaced by conducting substrates in inorganic Z-schemes to create ‘redox mediator-free’ schemes [2,4]. For example, Domen, Reisner and co-workers have realized a Z-scheme using a photoelectrochemical cell to produce formate with water as the electron source [52]. Their photocatalyst sheets are electrically connected and thus do not require redox mediators. Domen and co-workers have even connected carbon dioxide and water splitting on a single light-absorbing material Al-SrTiO_3_ [53]. However, as demonstrated by Domen and co-workers optimizing the conditions for these redox-mediator-free schemes is very different to optimizing particulate suspension Z-schemes [54].

The photocatalytic assemblies for Z-schemes are usually developed independently with different donors. Recently Ma et al. have developed a carbon dioxide reduction photosystem composed of a cobalt–quaterpyridine catalyst anchored to carbon nitride semiconductor particles [55]. Rather than an inorganic sacrificial donor, they developed this system using a benzimidazole sacrificial donor. As we have discussed, examples of recycling benzimidazoles already exist which makes them excellent candidates for donor recycling.

In contrast to those developed for water splitting, one of the first visible light-driven Z-scheme for carbon dioxide reduction consumed the sacrificial donor methanol to form formic acid and formaldehyde [56]. This system is interesting for a number of reasons. Rather than intermediate redox mediators shuttling charge between two photocatalytic assemblies, Ishitani, Domen, and co-workers covalently connected the catalytic systems and photosensitizers to enable direct electron transfer between them. In contrast to the system of Domen where the photoexcitation only occurs at the Al-SrTiO_3_ particle [53], the system developed by Ishitani included a second ruthenium chromophore linked the TaON particle to a ruthenium carbonyl catalyst [56]. This allowed the electron to be promoted by 2 photon absorption events, making it a Z-scheme. It can be argued that the linking ruthenium chromophore was acting as a redox mediator. Rather than using water as the sacrificial donor in this work, they used methanol which can be produced by photocatalytic or electrochemical carbon dioxide reduction. The oxidation product, formaldehyde, can be re-reduced. However, separation of formaldehyde and the carbon dioxide reduction product formic acid would be difficult. Therefore, a logical route for sustainably sourcing methanol would be using this system in combination with another that photo- or electrochemically reduces carbon dioxide to methanol.

### Recycling strategies

There are currently two favored methods emerging for sacrificial donor regeneration: photochemical recycling and electrochemical regeneration. The rehydrogenation of model compounds by thermochemical methods has also been demonstrated by Carpenter and co-workers [32]. The two clear strategies for recycling sacrificial donors are in-situ and ex-situ recycling. With the exception of existing Z-schemes and the ITIES water-splitting work, most sacrificial donor recycling methods are being developed separately from photocatalytic systems even if they are intended to be used for in-situ recycling*.* Although in-situ recycling is closer to actual photosynthesis, ex-situ recycling of sacrificial donors should be considered as another avenue to realize net artificial photosynthesis.

In-situ recycling of sacrificial donors and redox mediators would require less donor/mediator because the species would constantly be regenerated and not consumed. If the donor or mediator is stable under the reaction conditions, the cost and complexity of the donor can be higher than that of a sacrificial donor. However, it is likely that high steady-state concentrations of a donor will be needed and using the costs targets set for redox flow battery electrolytes is probably a sensible starting point. In an ideal system, the only inputs needed would be carbon dioxide, water, and light. This implies photochemical recycling in-situ. However, electrochemical in-situ recycling could be achieved in 2 ways: 1. The driving force for recycling an oxidized donor at an electrode could be supplied by a photosensitizer. 2. The driving force for donor re-reduction could be an applied voltage from renewable energy sources.

Following the example of ‘decoupled water splitting’ and redox flow batteries (RFBs) by recycling the electron donor ex-situ offers several potential advantages [8,57–58]. For example, decoupling carbon dioxide reduction and water oxidation in two separate reactors would allow the development of simpler chemical systems with less components and less factors to optimize per reactor. This would require less re-optimization of existing chemistry. Recyclable donors could be stored in tanks like RFB electrolytes, which decouples the 2 reactions and means rates do not have to be perfectly balanced [8]. Furthermore, photogenerated reactants could be stored in excess to keep any ‘dark’ processes running overnight. Schulz and co-workers reported a Cu(I) 4*H*-imidazolate photosensitizer that was reductively quenched in the presence of DMT to generate a stable reduced species [27]. The reduced species was so stable that it could be stored in the dark for hours and then be used to reduce methyl viologen. If DMT can be replaced with a more sustainable electron source, this could be part of a decoupled cycle either regenerating an oxidized sacrificial donor or using the reduced Cu(I) 4*H*-imidazolate itself as a donor. However, regenerated donors would need to be stored in large amounts to fuel a carbon dioxide reduction process at an industrially relevant rate. This means that they would have to be inexpensive, easy to synthesize, and made from earth abundant materials. They would also have to be stored in oxygen-free conditions to prevent re-oxidation.

A major advantage of ex-situ or decoupled sacrificial electron donor recycling is that, like decoupled water splitting, the gaseous products can be evolved separately preventing explosive mixtures. However, if the sacrificial donor needs to be separated from other homogenous catalysts or supporting electrolytes, this could be a challenge. Phase-separation systems such as the ITIES methods developed by Girault and Scanlon for water splitting could be a crucial starting point for developing scalable systems [35]. There is also a branch of artificial photosynthesis research investigating the compartmentalization of different reactions using liposomes and membranes [38]. Another alternative could be to use redox-active polymers as recyclable donors which would allow microporous membrane separation. Redox-active polymers are a very active area of research for aqueous and non-aqueous RFBs [59–60]. This is because often the most expensive component of an RFB is the ion-exchange membrane used to prevent the mixing of charged species and recombination. Microporous membranes are much less expensive, and the particulate size of redox-active polymers could be tuned to prevent unwanted crossover. Photosensitizers and catalysts have been successfully immobilized in polymeric matrices, which is also another approach to phase separation [61–62].

### Exploring candidates for recyclable electron donors

From our consideration of what makes an effective sacrificial electron donor we can highlight the chemical properties required by an effective recyclable electron donor or redox mediator. For a system that requires oxidative or reductive quenching, the oxidation potential of the donor must be less positive than the reduction potential of the oxidized photosensitizer or excited photosensitizer, respectively. The donor must be highly soluble, absorb light in a region that does not overlap with the absorption of the photosensitizer, and form a stable oxidation product that can be re-reduced in the presence of water. Redox mediators for in-situ recycling benefit from fast reversible electron transfer. However, if they are interacting with a molecular photosensitizer care needs to be taken to choose a species that maximizes cage escape yield and minimizes geminate recombination. Recyclable donors that must accumulate need to undergo electrochemically irreversible oxidation or an EC mechanism where the oxidized donor undergoes a chemical step that prevents recombination but forms a stable, recyclable byproduct. The dimerization of thiolates to disulfides is a good example because unlike dimers of NADH analogues that form carbon–carbon bonds the S–S bond is easily broken. The chemical step can also be a phase change as demonstrated in the work by Girault and co-workers [35]. Systems designed for PCET, whether excited-state PCET or PCET of the ground state species, will benefit from donors that carry a hydride or hydrogen atom equivalent rather than just electrons, as well as by carefully matching the oxidation potential of the donor to the redox potentials of the system. However, one must also carefully calculate the p*K*_a_ values of the system and choose a donor to match [17,45,49].

Measuring or calculating the redox potentials of photosensitizers is important for designing photocatalytic systems. Potential electron donors can be screened by comparing their oxidation potential to the redox potentials of the photosensitizer. Because redox potentials are solvent and pH-dependent, it is important to compare potentials recorded in conditions as close to the final catalysis conditions as possible. Once candidate donors have been identified using their oxidation potential, their quenching behavior and the effect on the photocatalytic performance can be tested.

**Figure 4:**
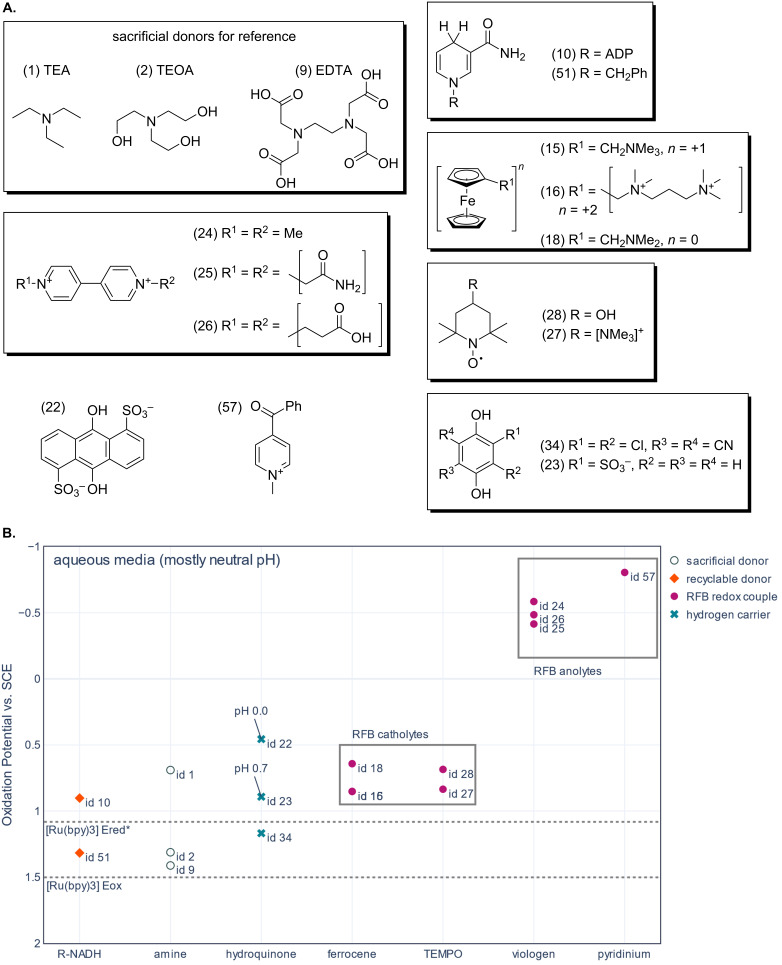
A. Structures of the molecules represented in part B. The numbers in brackets correspond to the compound id in Supporting Information File 1 and the data point labels in part B. B. Plot of oxidation potential vs SCE in aqueous media for compounds sorted by redox-active moiety. Note that the potentials are shown for the one electron-reduced viologen species, not the doubly reduced species. Dotted lines have been added to indicate the excited-state reduction potential (*E*_red_*) and oxidation potential (*E*_ox_) of ruthenium trisbipyridine chloride in aqueous media. Unless the data point is labelled, the potentials were recorded at neutral or unspecified pH. The references for the potentials can be found in the text and in Supporting Information File 1. The data point labels correspond to the compound id in Supporting Information File 1.

**Figure 5:**
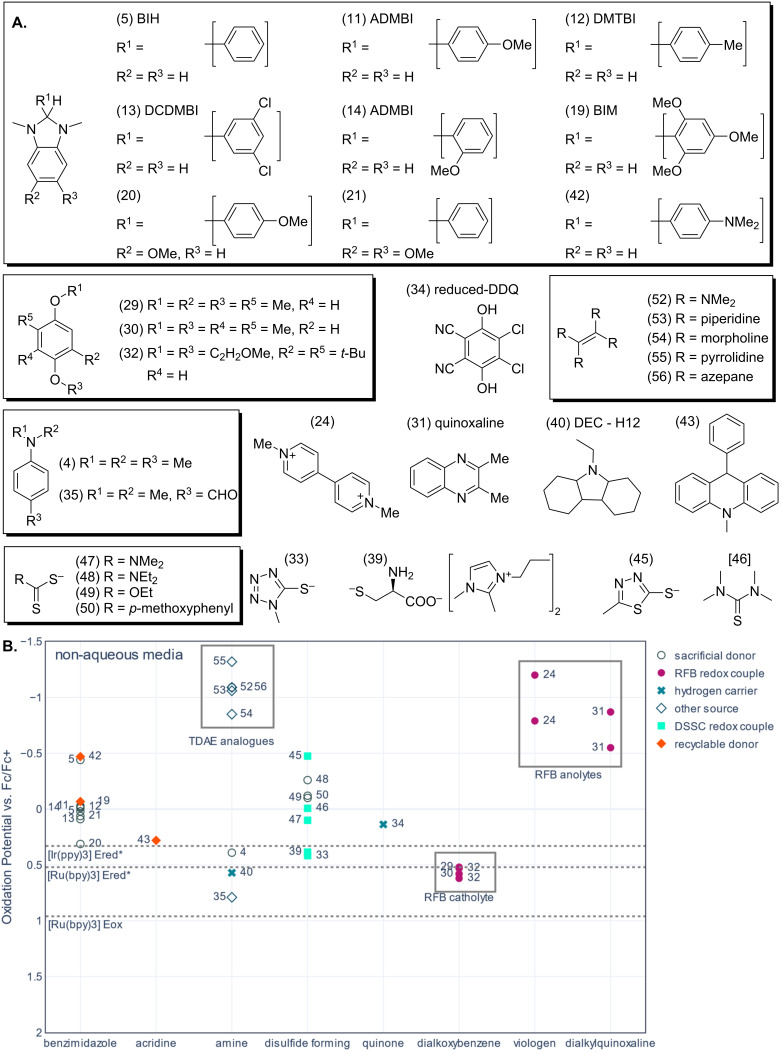
A. Structures of the molecules represented in part B. The numbers in brackets correspond to the compound id in Supporting Information File 1 and the data point labels in part B. B. Plot of oxidation potential vs Fc/Fc^+^ in non-aqueous media for compounds sorted by redox-active moiety. Note, that the potentials are shown for the dialkylquinoxaline example are the oxidation of the 1 and 2 electron-reduced species. Dotted lines have been added to indicate the excited-state reduction potential (*E*_red_*) and oxidation potential (*E*_ox_) of ruthenium trisbipy in acetonitrile aqueous media, as well as *E*_red_* of Ir(ppy)_3_ in DMF. The references for the potentials can be found in the text and in Supporting Information File 1, Table S2. The data point labels correspond to the compound id in Supporting Information File 1.

To help identify potential recyclable replacements for sacrificial donors such as triethylamine, the oxidation potentials of different families of electron donors have been plotted for both aqueous (Figure 4) and non-aqueous media (Figure 5). The oxidation and excited-state reduction potentials of ruthenium trisbipyridine (Ru(bpy)_3_) have been added to each figure. Ru(bpy)_3_ is a well-studied photosensitizer and the basis for many analogues used in artificial photosynthesis. This makes it a good compound to use as a guide for how low the oxidation potential of electron donor candidates needs to be. The oxidation potential of Ru(bpy)_3_ indicates the LUMO energy of the photooxidized sensitizer, and it is the relevant reduction potential for the regeneration of the photooxidized photosensitizer. The excited-state reduction potential of Ru(bpy)_3_ is the reduction potential relevant to reductive quenching. The potential axes on the graph have been inverted to aid the reader to visualize the electron transferring from higher energy electron donors to the lower energy levels of the photooxidized or photoexcited Ru(bpy)_3_. The data used to create the figures is available in the tables in Supporting Information File 1.

The examples of candidates for recyclable donors in aqueous systems shown in Figure 4 have been chosen from literature on redox flow batteries and decoupled hydrogen evolution, as well as photocatalysis. The oxidation and reduction potentials have been collected from various references and were converted to the potential scale vs SCE. Unless otherwise stated, the potentials were recorded in neutral conditions, or the pH was not specified.

The three amine examples, EDTA (1.41 V vs SCE), triethanolamine (1.31 V vs SCE), and triethylamine (0.69 V vs SCE), are all common sacrificial donors in photocatalysis. Although they cannot be regenerated, these examples indicate the operational potential range expected for photosensitizer quenching. Triethylamine has the lowest oxidation potential and is the most reducing of the 3 species (0.69 V vs SCE) which means that it can both reductively quench Ru(bpy)_3_ and regenerate photooxidized Ru(bpy)_3_ if the lifetime and electron-transfer rates are appropriate. EDTA has the highest reported oxidation potential and is the least reducing member of the amines shown (1.41 V vs SCE). Hence, it should only regenerate photooxidized Ru(bpy)_3_ in aqueous conditions. Unfortunately, Carpenter and co-workers did not measure the oxidation potential of their recyclable amine species for comparison [32].

The ferrocene, TEMPO, and viologen derivatives shown in Figure 4 are used in aqueous organic redox flow batteries [59,63]. The batteries store charge in concentrated aqueous solutions of small organic redox mediators that can be oxidized and re-reduced (or reduced and reoxidized) for 100s or 1000s of cycles. These redox mediators need to be highly soluble, stable, and their charged products need to be stable for days if not months. This makes them excellent candidates for redox mediators, or recyclable electron donors in aqueous artificial photosynthesis.

TEMPO and ferrocene derivatives are used to store positive charge in RFB catholytes [59,63]. Both parent compounds undergo reversible one-electron oxidation and re-reduction but had to be modified to improve their solubility. TEMPTMA and both ferrocene derivatives have one or more ammonium groups added to the core TEMPO or ferrocene charge-carrying moiety. This both increases the solubility of the species and the oxidation potential in aqueous media. TEMPOL uses an alcohol group to the same effect. Redox couples for RFB catholytes are optimized for increasing the oxidation which decreases their reduction capabilities. However, the TEMPO and ferrocene derivatives have oxidation potentials similar to triethylamine, which would be an ideal range to target. Although the oxidation potentials of the ferrocene and TEMPO derivatives are in an ideal range for electron donors, these compounds and their oxidation products are positively charged. This will stabilize the charge-transfer complex formed during quenching and combined with fast electron transfer will likely increase the rate of recombination and decrease their effectiveness as donors.

The oxidation potentials of the viologens (Figure 4) are shown for the one-electron-reduced viologen species ([methyl viologen]^•−^ −0.59 V vs SCE, [ethylcarboxy viologen]^•−^ −0.49 V vs SCE, [ethylamide viologen]^•−^ −0.42 V vs SCE) [59]. Viologens usually act as electron acceptors in aqueous RFB anolytes. In general, RFB anolytes are optimized to have very low reduction potentials. Once reduced, anolyte redox mediators become excellent reducing agents and viologens can be reduced twice in two separate one-electron transfer events. The viologen family of compounds has been the target of structural modifications to tune the redox potentials and enhance the solubility for RFBs [59]. Meanwhile, methyl viologen has been used as a sacrificial electron acceptor in photochemistry [27]. Therefore, viologen species could be used as redox mediators for linking water oxidation to carbon dioxide reduction by accepting electrons during water oxidation and donating them during carbon dioxide reduction to be reoxidized. Because viologens are positively charged and are reduced to neutral compounds, it is likely that the resulting charge-transfer complex will have a higher cage escape yield than a complex where the oxidized donor has a positive charge. However, in some rare cases the excessive driving force provided by reduced viologens (up to 1.28 V > triethylamine) might be too large a difference in energy and lead to Marcus inversion [64]. A more practical issue is the reoxidation of viologen in the presence of oxygen.

Redox flow battery anolyte research focuses on increasing solubility and stability while decreasing the reduction potential, weight, and cost of the anolyte species. To this end, Sanford and co-workers have worked on developing pyridinium analogues to out-perform the bipyridinium viologens [65–66]. The pyridinium analogue represented in Figure 4 can be reduced to a stable radical and reoxidized in aqueous media [65]. Depending on the pH, the reduction potential to form the 2 electron-reduced species is close to that of the stable radical. In non-aqueous media analogues of this species form stable 2 electron-reduction products. In aqueous media the 2 electron-reduced product can undergo side reactions. Interestingly at low pH the 2-electron product behaves like a redox catalyst for hydrogen evolution and can increase the pH from 4 to 11. Although this behavior is not desirable for RFBs it might be interesting for artificial photosynthesis. It is also noteworthy that the reduced pyridinium compounds resemble Hantzsch esters which are organic reductants commonly used in organic synthesis.

Quinones and hydroquinones have also been used in RFBs. Notably, 1,4-hydroquinone and 1,4-benzoquinone were used to create membrane-less RFBs with ITIES and charge-separation maintained in a flowing system [67–68]. Two of the examples shown in Figure 4 AQDS (0.46 V vs SCE at pH 0) and hydroquinone sulfate (0.89 V vs SCE at pH 0.7) have been used as hydrogen carriers in decoupled water splitting, rather than in RFBs [57]. Hydrogen carriers in decoupled water-splitting studies are re-reduced using protons from water in acidic conditions, and are used to transport hydrogen equivalents across membranes and between reactors. Some examples have been used as recyclable reductants [58]. These particular quinones have low enough oxidation potentials for reductive quenching of Ru(bpy)_3_ and reduction of photooxidized Ru(bpy)_3_. Furthermore, quinones have well-studied PCET chemistry [26]. 2,3-Dichloro-5,6-cyano-1,4,hydroquinone, the hydrogenated form of 2,3-dichloro-5,6-cyano-1,4-benzoquinone (DDQ), has the highest oxidation potential of the 3 quinone examples (1.17 V vs SCE). This means it does not have the energy to reductively quench Ru(bpy)_3_ [26]. Hydroquinones are a large family of compounds with a wide range of redox potentials and only a small sample has been shown here.

The NADH analogue BNAH which has been successfully regenerated using water is also shown in Figure 4. In aqueous media the oxidation potential of BNAH stays constant between pH 7 and 13 (if stable buffer is used) [69]. However, the oxidation potential increases and the reducing ability of BNAH and other NADH analogues decreases with pH after pH 7. NADH analogues are excellent candidates for electron donors in systems that require PCET because PCET prevents dimerizable radical formation. However, a too low pH value could be detrimental. The redox potentials recorded for BNAH were also reported to be very sensitive to the electrode material.

Many carbon dioxide reduction systems are being developed in non-aqueous solvents. Amines, disulfide-forming thiolates, and NADH derivatives, such as benzimidazoles and acridines, are used as sacrificial donors. Other families of compounds used in other applications such as non-aqueous RFBs, dye-sensitized solar cells, and LOHCs will also be discussed. The oxidation potentials of these compounds in non-aqueous media vs Fc/Fc^+^ are shown in Figure 5. The excited-state reduction potential of Ir(ppy)_3_ in DMF (V vs Fc/Fc^+^) has been added to the plot for comparison [70], as well as the redox potentials for Ru(bpy)_3_ in acetonitrile [20]. Ir(ppy)_3_ is a photosensitizer used for carbon dioxide reduction because it is one of the most photoreducing dyes available [71]. The redox potentials presented were recorded in a variety of non-aqueous solvents which means this graph is only a rough comparison. It is highly recommended that the oxidation potential of any compound of interest are re-recorded before using them to screen conditions for photocatalysis.

Although most benzimidazole examples have been marked as sacrificial donors in Figure 5, BIM (−0.07 V vs Fc/Fc^+^) and other analogues have been recycled by Glusac and co-workers [49]. It is highly likely that the same recycling methods could be extended to the rest of the benzimidazole family. Benzimidazoles are being adopted in organic photoreductions [30], and have been shown to increase the efficiency of carbon dioxide reduction systems using ruthenium-based photocatalytic systems [48,72]. This can be explained by the low oxidation potentials (0.31 to −0.47 V vs Fc/Fc^+^) amongst other factors. Bases are often added to enhance the quenching rate by deprotonation of benzimidazoles which means they are commonly used with TEA or TEOA [29–30]. Benzimidazoles are also one of the redox mediators that can be recycled photo- and electrochemically [45,49]. As illustrated in Figure 5, structural modification of the benzimidazole core alters the redox behavior and allows tuning of the oxidation potential. The benzimidazoles shown all have enough reducing power to reductively quench Ru(bpy)_3_ and possibly Ir(ppy)_3_. The tunability of benzimidazoles is particularly helpful when developing photocatalysis systems with new photosensitizers and their capability to undergo PCET in certain environments could be an advantage for some systems. Acridine compounds are also analogues of NADH and have electrochemical behavior amenable to recycling. The example shown has a redox potential that is more positive than most benzimidazoles but still suitable for the reductive quenching of Ru(bpy)_3_ (0.28 V vs Fc/Fc^+^) [45]. In aprotic media both benzimidazoles and acridines donate 2 electrons and a proton in separate redox events. However, only the oxidation potential to remove the first electron is shown for these species because it is this initial oxidation that generates the other electron-donating intermediates.

A variety of compounds have been grouped together with the amines label in Figure 5. 4-(*N*,*N*-Dimethylamino)toluene (DMT), has been used as a sacrificial electron donor in artificial photosynthesis [3]. The radical species that forms after oxidation can dimerize by forming a carbon–carbon bond which cannot be broken by re-reduction [3,73]. Voltammetric studies to identify the byproducts of DMT oxidation noted that the corresponding dimethyl amine – *p*-benzaldehyde oxidized at 0.79 V vs Fc/Fc^+^ and underwent a slow re-reduction [73]. Similar compounds, ketone derivatives rather than aldehyde, were developed with more negative potentials as anolytes for aqueous and non-aqueous RFBs by Sanford and co-workers [65–66]. The redox potential of the aldehyde is too positive for reductive quenching but low enough to regenerate photooxidized Ru(bpy)_3_. The aldehyde has been added to the figure because it is an interesting compound to consider when designing recyclable sacrificial donors, even though an example could not be found where it was used in artificial photosynthesis or RFB research.

The carbazole 9-ethyldodecahydro-1*H*-carbazole (DEC-H12) has an oxidation potential of 0.57 vs Fc/Fc^+^ and can carry 6 hydrogen equivalents [74] (DEC-H12 has been classified as an amine in Figure 5). The oxidation potential of DEC-H12 is lower than the oxidation potential of Ru(II)(bpy)_3_, which makes it a candidate for regenerating photooxidized Ru(bpy)_3_ in acetonitrile (depending on lifetimes etc.). However, the oxidation potential of DEC-H12 is not negative enough to allow reductive quenching. DEC-H12 has been studied in detail as a LOHC, which are small organic molecules that can be catalytically dehydrogenated and rehydrogenated [75]. Many other amines and organic molecules have been investigated as hydrogen carriers [40]. They mostly resemble traditional amine sacrificial electron donors but form more stable carbazole heterocyclic oxidation products [75]. LOHC compounds could potentially provide both protons and electrons for artificial photosynthesis. The thermodynamics of electrochemically hydrogenating several LOHCs using a modified water-splitting device have been investigated [40]. However, other electrochemical hydrogenation methods might be more appropriate [41–42].

There is already work on electrochemical dehydrogenation of LOHCs [76–77]. In one example, DDQ was used to remove hydrogen from secondary amines by oxidizing them, followed by reoxidation of the hydrogenated DDQ at the electrode to establish a redox catalysis cycle [76]. In non-aqueous media DDQ has a low oxidation potential (0.14 V vs Fc/Fc^+^ in acetonitrile) so that DDQ could potentially reductively quench Ir(ppy)_3_ and Ru(bpy)_3_ and regenerate the photooxidized species in acetonitrile [26]. However, in water the oxidation potential of DDQ (1.17 V vs SCE or 0.86 V vs Fc/Fc^+^) is too positive to thermodynamically allow quenching of Ru(bpy)_3_ [26]. This change in position and relationship between the various redox potentials is an illustrative example of the dramatic thermodynamic changes possible when moving from a non-aqueous system to an aqueous one. DDQ is also a particularly interesting example because it could potentially be used as an intermediate redox mediator to accumulate protons and electrons, or hydride equivalents from other donors. Similar schemes have been achieved using dithiols. However, the dithiols are usually attached to a photo- or electrocatalyst, and take advantage of potential inversion [78–79]. In the electrocatalytic study of DDQ one of the amines tested was successfully dehydrogenated in the presence of DDQ, yet in the absence of DDQ the amines generally formed polymeric byproducts under the same electrolysis conditions [76].

Another interesting family of amines are the tetraaminoethylene analogues (grey box in Figure 5). Tetra(dimethylamino)ethene (TDAE) is a strong one-electron reductant with a ground-state oxidation potential of −1.09 V vs Fc/Fc^+^ [80]. There are few organic electron donors with oxidation potentials this negative, except reduced RFB catholytes. However, TDAE has been used as a photocatalyst absorbing at 440 nm for dehalogenation which is within the visible region usually used by photosensitizers. It is also a very toxic, corrosive, and air-sensitive compound [80]. Hence, TDAE is not a good candidate for a recyclable donor. However, Charboneau et al. synthesized and reported a set of more stable analogues for use as reductants in catalysis for synthesis [81]. Tetrakis(*N*-pyrrolidinyl)ethylene (TPyE, −1.32 V vs Fc/Fc^+^) requires handling in a glovebox. However, 1,1,2,2-tetrapiperidinoethene (TPiE, −1.06 V vs Fc/Fc^+^) a piperidine analogue, TME (−0.85 V vs Fc/Fc^+^) a morphine analogue, and 1-[1,2,2-tris(azepan-1-yl)ethenyl]azepane (TAzE, −1.09 V vs Fc/Fc^+^) an aziridine analogue are all stable enough to handle at the lab bench. The visible absorption properties of these compounds were not reported but the more stable analogues may allow researchers to develop more reducing photosensitizers.

In contrast to aqueous organic RFBs, non-aqueous RFBs use small organic molecules that are highly soluble in non-aqueous media. Similar to their aqueous counterparts, redox mediators for organic RFBs are optimized to form stable charged intermediates by single-electron transfer reactions that can be charged and discharged over many cycles. Non-aqueous catholytes are also optimized to have more positive oxidation potentials and anolytes to have more extreme reduction potentials.

Sanford and co-workers used an innovative evolution strategy to engineer lightweight pyridinium anolytes to replace viologens in non-aqueous RFBs [66]. These species resemble oxidized Hantzsch esters but are stabilized in the *para* position. A similar pruning strategy was adopted by Huang et al. to create dialkoxybenzene anolytes for organic RFBs based on DBBB (2,5-di-*tert*-butyl-1,4-bis(2-methoxyethoxy)benzene) an alkylated quinone structure [82]. Hydroquinones and aqueous RFB anolytes have low enough potentials to be candidates for quenching Ru(bpy)_3_ in aqueous media. Dialkoxybenzene anolytes were developed from lithium ion battery chemistry and for non-aqueous RFBs as stable one-electron redox couples in lithium carbonate electrolytes [82–83]. The oxidation potentials recorded for a sample of these species range from 0.52 to 0.62 V vs Fc/Fc^+^. One compound, DBBB, has a 0.09 V shift in the oxidation potential recorded for two different carbonate solvents (0.53 V in EC/EMC and 0.62 V in PEC vs Fc/Fc^+^). These potentials are close to the excited-state reduction potential measured for Ru(bpy)_3_ in acetonitrile, however, the solvent dependence of the redox potentials means that it is not clear if the dialkoxybenzenes have enough reducing power for quenching ruthenium photosensitizers.

Dialkylquinoxalines were studied alongside DBBB as an alternative to viologens as doubly reducible anolytes for non-aqueous RFBs [83]. The two redox events shown in Figure 5 are two one-electron reduction potentials for the same dialkylquinoxaline species (−0.87 and −0.55 V vs Fc/Fc^+^). This example was the more electrochemically stable example in the study. Singly reduced viologens in water are at least 1.5 V more negative than the excited-state reduction potential of Ru(bpy)_3_, while in non-aqueous media dialkylquinoxalines appear closer to the excited-state reduction potential of Ru(bpy)_3_. However, like viologens, they are electron acceptors synthesized in their oxidized form and need to be reduced to be used as electron donors. This makes them excellent candidates for electrochemical or photochemical charging, perhaps even in two-phase systems to couple the charging to water oxidation. However, such species can also be reoxidized in the presence of oxygen which must be considered when designing an in*-*situ recycling system. For initial photocatalyst screening it is worth noting that reductants such as tetraaminoethene derivates or Hantzsch esters could be used to mimic the redox potentials of anolytes like quinoxalines. However, it would be better to generate the reduced viologens and quinoxalines to use for screening.

Thiolates such as ethylxanthate (−0.1 V vs Fc/Fc^+^), 4-methoxyphenylthioate (−0.12 V vs Fc/Fc^+^) and diethylthiocarbamic acid (−0.26 V vs Fc/Fc^+^) have been used as sacrificial electron donors in artificial photosynthesis and their very low oxidation potentials making them excellent reducing reagents [30]. Related thiazoles have been used in dye-sensitized solar cells as redox mediators because they oxidize to form a disulfide bridge that can be re-reduced [50,84]. These compounds seem like ideal candidates for recyclable electron donors because they exhibit an EC mechanism which results in an electrochemically recyclable dimer [50–51,84–85]. For instance, methylthiocarbamate (0.1 V vs Fc/Fc^+^), a redox mediator investigated for dye-sensitized solar cells, is a derivative of the sacrificial donor diethylthiocarbamic acid with only a slightly less driving force for the reductive quenching than the sacrificial donor [86]. Another redox mediator developed for dye-sensitized solar cells, 2-mercapto-5-methyl-1,3,4-thiadiazole has a much lower oxidation potential (−0.48 V vs Fc/Fc^+^) [87]. The work for dye-sensitized solar cells is particularly interesting because the thiols were developed as non-visible absorbing redox couples to replace colored I^−^/I_3_^−^ redox couple in dye-sensitized solar cell research [84]. They also span a wide potential range and mixing the thiolates to make mixed disulfides alters the oxidation potential of the mixture which offers an interesting optimization opportunity [85]. Cysteine has been used as a sacrificial donor and explored in non-aqueous dye-sensitized solar cells with an imidazolium cation (0.39 V vs Fc/Fc^+^) [51]. The resulting compound is similar in structure to an ionic liquid. In contrast, in artificial photosynthesis cysteine is often used in aqueous systems. Aromatic dithiols have been studied for accumulating charge by taking advantage of potential inversion, however, they often consume other thiols or thiolates to accumulate the charge [78–79]. For some of the thiolates herein the oxidation potential is reported rather than the standard potential because the peak-to-peak separation can be very large [85].

Finally, this is not an exhaustive list of candidates for recyclable donors. The field of RFBs, for instance, is constantly expanding the library of small organic redox mediators [88]. However, hopefully this discussion may act as a starting point for those interested in exploring the area.

## Conclusion

This review highlighted several species from different research fields with a range of appropriate oxidation potentials that can be recycled, either electrochemically or photochemically. This information will hopefully enable artificial photosynthesis researchers to continue to move away from unrecyclable sacrificial donors when developing their catalytic systems and utilize existing species that can be recycled, such as benzimidazole. However, oxidation potentials are only one property of recyclable electron donors that need to be optimized. More small organic recyclable electron donors need to be designed with economic and sustainable syntheses. They need to be highly soluble and possess modifiable, modular structures that can be used to simply tune the redox potentials, quenching rates, and p*K*_a_H to match different dyes and quenching pathways. Finally, although recyclable donors such as benzimidazoles are being used in developing new carbon dioxide reduction catalysts, more proof-of-concept studies are required to demonstrate combining photocatalysis with sacrificial donor recycling.

## Commonly used Abbreviations and Symbols

**Table 1:** Explanations of abbreviations.

Abbreviation	Explanation

BIM	1,3-dimethyl-2-(2,4,6-trimethoxyphenyl)-2*H*-benzimidazole
DEC-H12	9-ethyldodecahydro-1*H*-carbazole
DcMFc	decamethylferrocene
ITIES	interface between two immiscible electrolyte solutions
LOHC	liquid organic hydrogen carrier
PCET	proton-coupled electron transfer
RFB	redox flow battery
Ru(bpy)_3_	ruthenium trisbipyridine cation in varying oxidation states

## Supporting Information

Link to a github code repository containing redox potential data collected from various references and the code used to create the plots of oxidation potential https://github.com/glowe691/redox_donors_notebooks/tree/submitted_belstein_JOC_for_peer_review .

File 1 A spreadsheet containing the tables with the oxidation potential data for the electron donors and redox active compounds discussed in this review. The table contains DOI numbers for the references where the potentials were reported.
